# Exploring *Toxoplasma gondii*´s Biology within the Intestinal Epithelium: intestinal-derived models to unravel sexual differentiation

**DOI:** 10.3389/fcimb.2023.1134471

**Published:** 2023-05-29

**Authors:** Florencia Sena, Saira Cancela, Mariela Bollati-Fogolín, Romina Pagotto, María E. Francia

**Affiliations:** ^1^ Laboratory of Apicomplexan Biology, Institut Pasteur Montevideo, Montevideo, Uruguay; ^2^ Laboratorio de Bioquímica, Departamento de Biología Vegetal, Universidad de la República, Montevideo, Uruguay; ^3^ Cell Biology Unit, Institut Pasteur Montevideo, Montevideo, Uruguay; ^4^ Molecular, Cellular, and Animal Technology Program (ProTeMCA), Institut Pasteur Montevideo, Montevideo, Uruguay; ^5^ Departamento de Parasitología y Micología, Facultad de Medicina, Universidad de la República, Montevideo, Uruguay

**Keywords:** *Toxoplasma gondii*, sexual differentiation, felinization, intestine, *in vitro* models, *ex vivo models*

## Abstract

A variety of intestinal-derived culture systems have been developed to mimic *in vivo* cell behavior and organization, incorporating different tissue and microenvironmental elements. Great insight into the biology of the causative agent of toxoplasmosis, Toxoplasma gondii, has been attained by using diverse *in vitro* cellular models. Nonetheless, there are still processes key to its transmission and persistence which remain to be elucidated, such as the mechanisms underlying its systemic dissemination and sexual differentiation both of which occur at the intestinal level. Because this event occurs in a complex and specific cellular environment (the intestine upon ingestion of infective forms, and the feline intestine, respectively), traditional reductionist *in vitro* cellular models fail to recreate conditions resembling *in vivo* physiology. The development of new biomaterials and the advances in cell culture knowledge have opened the door to a next generation of more physiologically relevant cellular models. Among them, organoids have become a valuable tool for unmasking the underlying mechanism involved in *T. gondii* sexual differentiation. Murine-derived intestinal organoids mimicking the biochemistry of the feline intestine have allowed the generation of pre-sexual and sexual stages of *T. gondii* for the first time *in vitro*, opening a window of opportunity to tackling these stages by “felinizing” a wide variety of animal cell cultures. Here, we reviewed intestinal *in vitro* and ex vivo models and discussed their strengths and limitations in the context of a quest for faithful models to *in vitro* emulate the biology of the enteric stages of *T. gondii*.

## 1 Introduction

Apicomplexans make up a large phylum of parasites characterized by the presence of an apical complex of secretory organelles which allows them to interact with, and invade, their host cell of preference. Beyond the conservation of these organelles, apicomplexan parasites differ greatly in their biology, including the range of host species, and even the cell type they invade within a host (Dubey, 2009). *Toxoplasma gondii*, the causative agent of toxoplasmosis, is arguably one of the most promiscuous parasites within the phylum in terms of host range and cell type preferences. Its multiple routes of infection, ample host cell range (virtually any warm-blooded species), host cell invasion capacity (virtually any nucleated cell), and capacity to chronically persist, render it one of the most successful zoonotic parasites of humans and animals worldwide (Flegr et al., 2014).

**Figure 1:**
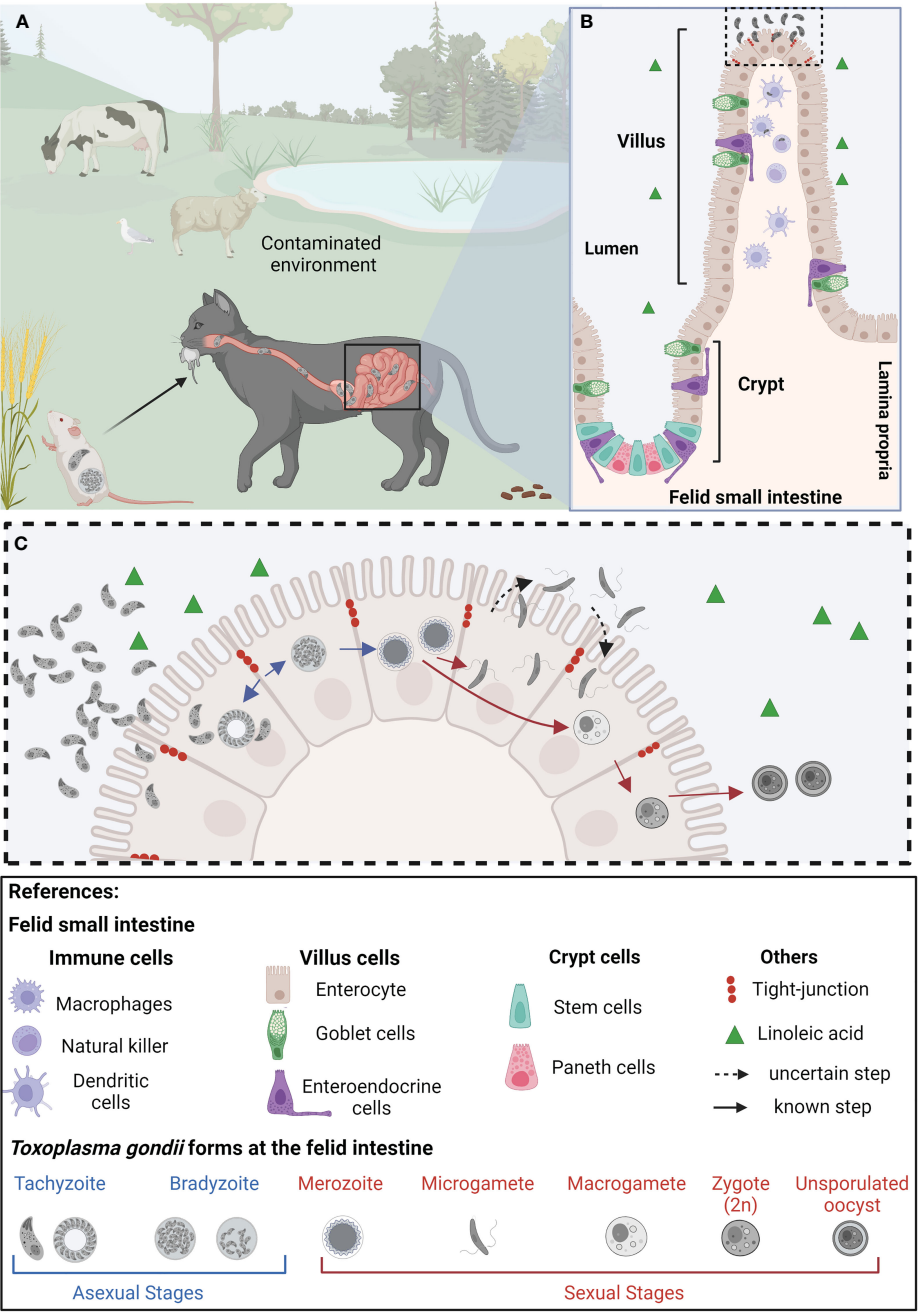
Representation of the life cycle of *Toxoplasma gondii* in epithelial cells of the small intestine of felids. **(A)** Felids acquire *Toxoplasma gondii* through consumption of contaminated food and/or water. Once in the small intestine’s epithelium, tachyzoites replicate and differentiate into bradyzoites. **(B)** The felid small intestinal epithelium is composed of diverse specialized cell types, all of them originating from intestinal stem cells, located at the base of the crypts. The feline intestinal environment, characterized by the absence of the delta-6-desaturase enzyme, and a consequent increased level of linoleic acid, is the only anatomical site and biochemical environment supporting *T. gondii’s* sexual differentiation **(C)** In the epithelial cells, *T. gondii* bradyzoites (haploid cell; 1n) differentiate into merozoites (1n), which further give rise to microgametes (1n) and macrogametes (1n). Through mechanisms not yet completely understood, the microgamete fertilizes the macrogamete generating the zygote (immature oocyst, 2n). These diploid cells are then shed as unsporulated oocysts in the felid’s feces, contaminating plants and water sources in the environment, becoming potentially infective to other animals, leading to the beginning of a new infective cycle. Created with BioRender.com.

*T. gondii* can be transmitted among animals through the carnivory of persistent cysts lodged in skeletal muscle or brain tissue. Likewise, the parasite is able to cross the placenta and infect the developing fetus if acquired during pregnancy. Finally, it can be ingested from the environment in the form of environmentally resistant oocysts, shed by felids in their feces (Dubey, 2006; Arranz-Solís et al., 2021). The best-studied life stage of the parasite is called the tachyzoite. This fast-replicating form of the parasite is responsible for acute toxoplasmosis. Tachyzoites can be readily grown and maintained *in vitro*, and a plethora of tools for their genetic manipulation have been developed. Though acute infection is the most clinically relevant, the vast majority of acute toxoplasmosis goes unnoticed in immunocompetent individuals quickly turning into latent chronic infections (Pittman and Knoll, 2015). Chronic stages arise by a switch in parasite metabolism to the slow replicating, latent, bradyzoite form. Bradyzoites persist in immune-privileged anatomical sites, such as the brain and the eye, and within skeletal muscle. However, bradyzoites can reactivate if the immune pressure decays. Multiple iterations of reactivation of latent bradyzoites in the eye cause progressive eye loss, even in immunocompetent patients (Sullivan and Jeffers, 2012). Carnivorism of bradyzoites infected tissue leads to their reactivation in the host’s intestine, and the systemic dissemination of tachyzoites, thereby closing the cycle of transmission among intermediate hosts (Cerutti et al., 2020). On the other hand, when a wild or domestic felid consumes bradyzoite-infected tissue or an oocyst from the environment, the parasite initiates its sexual differentiation cycle within the cat’s intestinal epithelium. Pre-sexual stages of the parasite include merozoites and gamonts, which eventually lead to the formation of fully differentiated gametes. Gametes can sexually recombine, forming oocyst precursor stages, which eventually lead to the shedding of environmentally resistant unsporulated oocyst. Environmental exposure to oxygen induces sporulation, leading to infective oocysts loaded with sporozoites which can persist in the environment for years (Ferguson, 2002) (**Figure 1A**).

*In vitro* modeling of the life stages of *T. gondii* has been traditionally limited to 2D cultures whereby the tachyzoite form expands quickly and efficiently, allowing for the generation of large amounts of material for different analyses. This has served to study genome modifications, gene expression control, the parasite’s kinome, secretome, proteome, phospho-proteome and genome-wide gene essentiality, among others, using tachyzoites. Despite the importance of persistence of the chronic forms and the role played by cats in the dissemination of *T. gondii*, our understanding of these aspects of parasitic life is limited. Their anatomical sequestration to inaccessible sites such as the brain and eye, and the lack of *in vitro* models to recreate some life stages *ex vivo* challenge our access to their biology. In particular, the interplay among tachyzoites, bradyzoites, and host factors, in the context of stage transitions within the intestinal epithelium cannot thus far be mimicked in traditional 2D cultures. The study of these aspects of parasite biology has traditionally relied on animal models, encompassing a number of unavoidable experimental limitations. However, recent technological breakthroughs in 2D and 3D culture systems provide promising routes for exploring aspects of parasitic life traditionally inaccessible. Herein, we review the state of the art in *in vitro* intestinal models and highlight their potential applications for characterizing different life forms of *T. gondii* within the enteric epithelium. We focus on the challenges and experimental opportunities offered by these up-and-coming experimental platforms for studying the sexual stages of *T. gondii*.

## 2 Intestine structure

The small intestine, part of the gastrointestinal system, is divided into three sections: duodenum, jejunum, and ileum (Carr and Toner, 1984). The intestinal mucosa comprises the epithelium, the underlying lamina propria, and a thin muscle layer called muscularis mucosa. Together, the lamina propria and the intestinal epithelium are organized into finger-like protrusions known as villi, interspaced by pocket-like invaginations called crypts. The crypt contains intestinal stem cells (ISCs) and Paneth cells characterized by the presence of dense granules containing antimicrobial peptides (AMPs). Paneth cells are interspersed with the ISCs contributing together to the intestinal stem cell niche (Antfolk and Jensen, 2020). The villi contain differentiated cell types collectively known as intestinal epithelial cells (IECs). IECs are frequently replaced by intestinal epithelial stem cells originating at the bottom of crypts, which differentiate along the villus in the so-called crypt-villus axis. Goblet cells provide a protective barrier and help lubricate the inner wall of the intestine layer by producing mucus over the intestinal epithelium. Finally, enteroendocrine cells carry out endocrine functions (Hewes et al., 2020). Additionally, the intestinal epithelium permanently interplays with the immune system; approximately 70% of immune cells are in the gut (Wiertsema et al., 2021).

## 3 *Toxoplasma gondii* within the intestine

Upon oral infection *via* carnivory or interaction with environmental sporulated oocysts (**Figure 1A**), parasites enter the gastrointestinal system of both intermediate and definitive hosts within days. The cyst wall protects them from the acidic gastric pH and ensures the passage to the small intestine where they excyst upon contact with bile salts and trypsin (Dubey et al., 1998). Parasite invasion and replication takes place at the intestinal villi and subsequently tachyzoites are released into the intestinal lumen, able to invade neighboring villi cells and to disseminate within the host *via* the lamina propria (Dubey, 1997). *T. gondii* dissemination across the different biologic barriers of the gastrointestinal tract requires the activation of specific invasion, attachment, and transmigration mechanisms which fast-tracks their dissemination to different tissues, such as lymph nodes, heart, eye and brain (Jones et al., 2017).

The initial steps of *T. gondii* infection within the gut rely on its ability to rapidly cross the epithelial barrier of the small intestine (**Figure 1B**). Astonishingly, this process takes place in a matter of seconds (Jones et al., 2017). Strategies used by *T. gondii* to cross the intestinal epithelium and reach the lamina propria include invasion of the intestinal epithelial cells, transepithelial migration, or by means of a “Trojan horse” mechanism, whereby *T. gondii* hijacks diverse immune cells to go undercover (Dobrowolski and Sibley, 1996; Barragan et al., 2005; Gregg et al., 2013). Parasites actively invade a wide range of cells including intestinal as well as immune cells and undergo an intracellular asexual lytic cycle of intracellular growth and multiplication before rupturing the host cells (Black and Boothroyd, 2000).

Release of newly formed tachyzoites into both the intestinal lumen and underlying tissues of the lamina propria, activate an acute immune response by secretion of a host of inflammatory factors by intestinal tissue-resident cells (Pittman and Knoll, 2015). The recognition of *T. gondii* by cellular innate sensors is the first line of host defense triggering the production of proinflammatory cytokines such as IL-1β and TNF-α by macrophages, neutrophils, and dendritic cells (Coombes et al., 2013; Sasai and Yamamoto, 2019). Altogether, these play a crucial role in the activation of the host immune responses which might ultimately trigger the switch from tachyzoite to bradyzoite (Cerutti et al., 2020).

### 3.1 Sexual differentiation of *Toxoplasma gondii*

Members of the *Felidae* family act as the only definitive hosts of *T. gondii* being responsible for its horizontal transmission through the distribution of infective oocysts released in their feces (Dubey, 2006). *Via* carnivorism, bradyzoites access the feline intestinal epithelium where they differentiate into merozoites, initiating the parasite’s sexual differentiation track (Weiss, 2000; Dubey, 2006). Sexual differentiation encompasses gametogenesis which implies the formation of macro (♀) and microgametes (♂). Their fusion generates diploid zygotes that eventually encyst and are shed in cat’s feces as immature oocysts sporulating and becoming infective in the environment (Sibley et al., 2009).

Within the feline intestine bradyzoites turn into merozoites, initiating sexual differentiation. For fertilization to take place, microgametes generated within the feline small intestine swim through the intestinal lumen to find a host cell containing a macrogamete (Ferguson, 2002) (**Figure 1C**). Ultimately, oocyst formation depends on microgamete motility; in turn, their ability to move lies in their ultrastructure. The ultrastructure of the sexual stages was well documented by electron microscopy studies over 50 years ago, studying small intestine of cats orally infected with *T. gondii* cysts (Pelster and Piekarski, 1971; Dubey and Frenkeñ, 1972; Scholtyseck et al., 1972; Ferguson et al., 1975). The stages of *T. gondii* giving rise to sexual forms differ from each other regarding the number of apical organelles, the shape and electron density of the rhoptries, the location of the nucleus, and the presence or absence of polysaccharide granules (Ferguson and Dubremetz, 2020). The merozoite becomes more spherical and loses the majority of its apical organelles, such as the rhoptries and dense granules, and appears to increase the size of its mitochondrion, which locates at the cell periphery. Sexual-specific organelles are unique signatures of sexual forms of *T. gondii*. Microgametes have an ellipsoid-shaped morphology with two motile flagella per cell assembled at the microgamete´s apex. During differentiation to microgametes, the parasite undergoes schizogony, a cell division mechanism that generates a syncytium-like cell bearing multiple nuclei. Nuclei move to the periphery of the cell with two centrioles, which presumably become basal bodies of the developing flagella. Flagella grow by protruding out into the parasitophorous vacuole. The flagellar axonemes are canonical in ultrastructure, being composed of nine microtubule duplets and a central pair (9 + 2), similar to what is observed in other apicomplexans (Ferguson, 2002; Morrissette and Sibley, 2002; Francia et al., 2016; Tomasina and Francia, 2020). On the other hand, macrogametes have an oval shape and contain numerous electron dense structures within the cytosol named “wall forming bodies.” During the development of the macrogamete there exists an increment in the size of the peripherally mitochondrion and the centrally located apicoplast. These changes are accompanied by the surge of an enlarged nucleus showcasing dispersed chromatin and a large nucleolus, with no accompanying nuclear mitosis (Ferguson and Dubremetz, 2020). The wall forming bodies contain polysaccharide granules, lipid droplets, and protein-rich wall forming bodies type W1 and W2 that will contribute to formation of the macrogamete (Freppel et al., 2019). Later on, these will play a crucial role in the formation of the oocyst wall (Ferguson et al., 1975; Ferguson and Dubremetz, 2020).

The study and identification of sexual differentiation components of coccidia parasites could powerfully impact our ability to design transmission-blocking strategies. For example, vaccines incorporating antigens from sexual stages could reduce oocyst formation (Cruz-Bustos et al., 2022). Genetic admixing can occur only if two genetically different strains coexist in the epithelium of the same feline. Therefore, fertilization is key to the generation of natural genetic diversity as this is the only phase in which the parasite exists in a transient state of diploidy. A recent study of 156 distinct parasite genomes present worldwide, concluded that the evolution of unique haplotypes of *T. gondii* generated by sexual admixing in cats, accompanied the evolution of felids from wild to domestic, and the family’s expansion in the last five centuries to the Americas (Galal et al., 2022), illustrating the importance of this process. Moreover, understanding the molecular underpinnings of gametogenesis and gamete fusion, could open up the possibility of *in vitro* controlling sexual admixing, opening new avenues for the generation of hybrid strains with biotechnological potential.

The lack of convenient experimental models has long stymied molecular studies of sexual stages of coccidian parasites. The detailed cellular mechanisms governing differentiation, gamete fusion and fertilization have only recently begun to be clarified. Gene transcription analyses of the enteric stages of *T. gondii* could allow the identification of genes expressed in sexual stages providing the potential of being *in vitro* engineered stage conversion by modulating gene expression (Reviewed in Ramakrishnan and Smith, 2021). Thus far, transcriptomic analyses have relied on *in vivo* generated sexual stages in orally infected felids. While the temporal resolution of these studies is hindered by the natural kinetics of differentiation whereby multiple stages are present at the gut at once, numerous important factors have been put forward as putative controllers of the differentiation process and as essential for fertilization. For example, the expression of the male gamete fusion factor HAP2 has been described as a key factor for *T. gondii* fertilization to take place (Ramakrishnan et al., 2019). This had been previously established for the ortholog in the malaria parasites whereby HAP2 was shown to be essential for fusion of gametes (Liu et al., 2008; Angrisano et al., 2017). *T. gondii* parasites lacking the microgamete-specific gamete fusion protein HAP2 fail at completing fertilization, undergo meiosis and consequently produce aberrant oocysts *in vivo* (Ramakrishnan et al., 2019).

The identification of the key transcription factors controlling gene expression components related to sexual commitment provides an overarching view of the regulatory networks underlying differentiation. A single validated class of apicomplexan transcription factors, the *apetalla* family of DNA binding proteins (ApiAP2s), has been extensively investigated as potential regulators and mediators of parasite differentiation (Sharma et al., 2020). In addition, microorchidia (MORC) was shown to complex with multiple AP2 transcription factors and the lysine deacetylase HDAC3 whose transcriptional control mediates transitions between asexual forms and also the onset of sexual differentiation. Remarkably, conditional silencing of MORC triggers the differentiation of *T. gondii* tachyzoites, in a 2D culture, into bradyzoites, merozoites, and gametocytes (Farhat et al., 2020). Recently, transcription factors from the AP2 family have been described to be implicated in silencing genes necessary for merozoites conversion and development of stages critical for sexual commitment (Antunes et al., 2023; Srivastava et al., 2023). A family member of AP2 transcription factors (AP2XII-2) has been identified to coordinate the recruitment of HDAC3/MORC complex to repress developmentally-controlled genes, such as the AP2X-10 (an oocyst specific AP2) and AAH1 (involved in oocyst maturation) (Srivastava et al., 2023). In addition, AP2XI-2 and AP2XII-1 heterodimers restrict the accessibility of chromatin to the transcriptional machinery by the recruitment of MORC and HDAC3 at merozoite promoter genes (Antunes et al., 2023). Finally, the post-translational N-glycosylation of multiple *T. gondii* proteins at the small intestine of cats orally infected with cysts has been documented (Zhai et al., 2022). However, how these modifications contribute to the sexual stages of *T. gondii* at the small intestine of felids remains unclear.

## 4 Intestinal *in vitro* and *ex vivo* models to study *T. gondii* biology

Despite its inherent importance and its potential for contributing to prophylactic strategies and biotechnology, little is known about the basic aspects of the mechanisms and biology of sexual differentiation in *T. gondii*. Traditionally, the study of this stage was ethically and technically hampered by the imperative need to use felid animal models. However, enteric cells of cats have arisen as a unique system to study the processes of sexual differentiation of *T. gondii.* In addition, a recent methodological breakthrough identified the biochemical fingerprint that triggers the differentiation of *T. gondii* to its sexual forms in the feline intestine. The study deciphered that a lack of delta-6-desaturase activity, exclusively lacking in the small intestines of cats, and the subsequent accumulation of linoleic acid, are sufficient to trigger the differentiation of *T. gondii* into its sexual stages (Di Genova et al., 2019). Remarkably, the use of a specific inhibitor of the delta-6-desaturase enzyme in mouse intestinal organoid derived monolayers infected with *T*. *gondii* enhanced the progression through the sexual stages (Di Genova et al., 2019). Furthermore, infected mice treated with a delta-6-desaturase inhibitor and fed a diet rich in linoleic acid resulted in meronts and gametes differentiation leading to infective oocyst shedding in their faces. These findings now allow the potential of “felinizing” 2D cultures of diverse, non-felid derived, origins. However, oocyst production is the ultimate indicator of efficient parasite sex; despite the fact that the feline enteric environment can be chemically mimicked *in vitro*, generating *T. gondii* sexual stages, fertilization does not take place. It is likely that additional factors are required for successful fertilization and oocyst production.

Below we contextualize the use of model systems in increasing order of model complexity (in terms of number of cellular types present, architecture and integration of physiological features) to study *T. gondii*´s biology within the intestine, particularly focusing on sexual differentiation.

### 4.1 Cell cultures from *Felidae* origin

Two dimensions (2D) cell culture is extremely useful in cell biology and has been widely implemented for the growth and propagation of different apicomplexan parasites (Ramírez-Flores et al., 2022). Traditionally, 2D cultures have served as powerful *in vitro* tools to study invasion, replication, and egress processes, profoundly impacting our understanding of these critical aspects of intracellular parasite life.

The establishment of an optimized system to grow primary Feline Epithelial Intestinal Cells (FEIC) and propagate them *in vitro* was successfully described (Zhao et al., 2018). Prior to this, primary cultures of FEIC obtained from the jejunum-ileum region had been explored as a tool to investigate the sexual differentiation of *T. gondii*, by infecting them with bradyzoites. The development of the syncytial-like forms of *T. gondii* were observed after 6 hours of infection, suggesting the presence of sexual stages of the parasite by light microscopy (Moura et al., 2009). Despite this, the use of FEICs monolayers has not been extensively adopted in the culturing of *T. gondii* most likely because of the complexity to obtain, propagate and preserve these cells (de Muno et al., 2014). In addition, there are ethical concerns with respect to the use of felids as experimental models.

Our understanding of the signals triggering sexual differentiation has exponentially increased. This is providing researchers with the opportunity to genetically manipulate the parasite factors that repress expression of sexual differentiation specific genes. In addition, mimicking the biochemical environment of the felid’s intestine, in any intestinal-derived *in vitro* model system is now also possible. This remarkable breakthrough provides an unprecedented opportunity to address biological questions of *T. gondii* gametogenesis that were inaccessible before without resorting to FEIC, expanding the breadth of cell lines that could be used to mimic these conditions in 2D cultures. In the next section we highlight the models based on cell lines not derived from felids, and their potential contribution to investigating both the mechanisms of intestinal pathogenesis and dissemination, and the complete sexual cycle of *T. gondii* by means of exogenous “felinization” (**Table 1**).

**Table 1:** Epithelial intestinal cells used for studying *T. gondii* and related coccidian parasite biology.

Cell culture model		Origin	Model Complexity Features		Applications	Reference
			Spatial organization	Fluidics		
immortalized cell line	Caco-2	human colorectal adenocarcinoma-derived	2D/3D	no	invasion mechanisms	(Briceño et al., 2016; DeCicco RePass et al., 2017).
	T84	transplantable human carcinoma cell line derived from a lung metastasis of a colon carcinoma	2D	no	electrophysiology/ion transport	(Di Genova and Tonelli, 2016)
	HT-29	human colorectal adenocarcinoma cells	2D/3D	no	ion transport/parasite multiplication	(Kowalik et al., 2004; Monroy, 2008; Cardenas et al., 2020)
	SW-480	large intestine human colorectal cancer	2D	no	parasite multiplication	(Majumdar et al., 2019)
primary cells	mICc12	mouse intestinal epithelium	2D	no	innate intestinal responses	(Mennechet et al., 2002; Gopal et al., 2011)
	IEC-6	rat small intestine- derived epithelium	2D	no	parasite invasion and multiplication	(Dimier and Bout, 1993; Weight et al., 2015)
	FHs 74 Int	human small intestine	2D	no	parasite proliferation	(Quan et al., 2018)
	FEIC	feline epithelial intestinal cells	2D	no	sexual differentiation	(Moura et al., 2009; Zhao et al., 2018)
	MODE-K	murine intestinal epithelial cells	2D	no	epithelial cell responses	(Johnson et al., 2014)
stem-cell derived	HOPX+	pig intestinal stem cells from crypts and treated with adenovirus	2D	no	intestine mucosa responses	(Stewart et al., 2021)
	air-liquid interface (ALI)	mouse intestinal epithelial stem cells and fibroblast	2D	no	life cycle development	(Wilke et al., 2019)
	collagen-supported epithelial sheet	mouse jejunal and ileal tissues	2D/3D	no	parasite-intestinal epithelium interaction	(Luu et al., 2019)
	organoids	livestock proximal jejunum	2D/3D	no	long-term renewable system for infection	(Derricott et al., 2019; Hares et al., 2020)
		duodenal sections from human, chicken, mouse, and porcine	2D	no	parasite-host interactions and parasite co-infections	(Holthaus et al., 2021)
		mouse proximal small intestine	2D/3D	no	immune responses	(Araujo et al., 2021)
		mouse small intestine	2D/3D	no	infection mechanisms	(Betancourt-Delgado et al., 2019)
		human small intestine	2D/3D	no	immune host responses	(Humayun et al., 2022)
		felid fetal small intestine (jejunum)	2D/3D	no	sexual differentiation	(Di Genova et al., 2019)
intestine on-a-chip	human intestinal microphysiological system	human jejunal tissue	3D	yes	innate immune cell responses	(Humayun et al., 2022)
explants	colonic explants	adult murine colon	3D	yes	long-term parasite infection	(Baydoun et al., 2017)

### 4.2 Human intestine-derived cell lines from immortalized, primary culture, and stem cells

Immortalized cell lines have been manipulated to proliferate indefinitely and can be cultured for long periods of time. A large number of such human intestinal cell lines have been established, such as, the well-known human colorectal adenocarcinoma-derived cell lines HT-29 and Caco-2. Human colon carcinoma-derived cell lines vary extensively in their degree of differentiation, proliferation, and metabolic properties (Rousset, 1986). Some of these cell lines are able to express differentiation features characteristic of mature intestinal cells, under certain conditions. Thus, Caco-2 cells, originally non polarized cells, can spontaneously differentiate into mature enterocytes with a basal and apical compartment clearly defined when seeded on filter inserts and culture for two to three weeks (Ferruzza et al., 2012). Under these culture conditions Caco-2 cells better recreate enterocyte morphology, with well-developed microvilli on the apical side, tight junctions between cells, and expression of specific hydrolase enzymes at the apical membrane (Sambuy et al., 2005; Verhoeckx, 2015).

*T. gondii* has successfully infected diverse intestinal cell lines, albeit only replicating asexually. Nevertheless, a bulk of studies have contributed to not only deciphering the mechanisms of *T. gondii* infection of enteroepithelial cells, but have also provided insight into the genetic, cellular, and biochemical mechanisms used by the parasite for dissemination. Caco-2 has been the most widely immortalized cell line used to model the infection of *T. gondii* in the enteric environment. Studies of *T. gondii* infection in Caco-2 cells have determined that infection leads to the loss of integrity of intestinal mucosa by increased paracellular permeability, reduction of transepithelial electrical resistance, loss of cytoskeleton organization, and redistribution of tight junction proteins as strategies to improve invasion (Barragan et al., 2005; Briceño et al., 2016; Ross et al., 2019).

Tight junctions (TJ) are selective gates that control paracellular diffusion of ions and solutes between cells (Zihni et al., 2016). Several enteric pathogens disrupt the TJ of intestinal epithelial cells as an infection strategy, and they have different ways to disrupt them. Tight junction proteins, such as occludin, claudins, zonula occludens (ZOs), and Ig-domain adhesion proteins (IgCAMs) are frequent targets of intestinal pathogens in the process of invasion and infection (Barragan et al., 2005; Briceño et al., 2016). In addition, another strategy to affect the TJ is the alteration of the cytoskeleton which serves to stabilize the TJ structure (Berkes et al., 2003). Overall, *T. gondii* infection transiently alters de TJ stability in Caco-2 cells to facilitate transmigration. The mechanism(s) used by *T. gondii* to interfere with the TJ in the host cell remains to be determined in detail. However, the increase of the permeability of intestinal TJ barrier by *T. gondii* in another immortalized culture cell called T84 (from transplantable human carcinoma cell line of colon) and in Caco-2 have been described to be modulated by proinflammatory cytokines such as IFN-γ or IL-1β from the host cell (Al-Sadi and Ma, 2007; Boivin et al., 2009).

Additionally, SW-480 cells established from colon-derived primary adenocarcinomas and Caco-2 have been used as a model to study parasite multiplication within the host cell. Parasites modulate different host cell pathways to promote their replication. The function of the β-catenin pathway, an intracellular signal transducer in the Wnt signaling pathway, regulates innate immunity for the benefit of parasite multiplication (Majumdar et al., 2019) as well as the early expression of beta-defensin 2 used as a mechanism from the parasite to modulate immune evasion in infected IECs (Morampudi et al., 2011). These studies have highlighted the cross-talk between the host immune system and the mechanisms of invasion and multiplication of *T. gondii*. In this context, defense mechanisms from the host cell using these cell models has been addressed. *T. gondii* tachyzoites successfully invade human colon-derived carcinoma HT29/B6 cells. The infection of HT-29 cells results in high expression of host 14-3-3 proteins as a possible strategy to increase cell survival and may prevent parasite replication (Monroy, 2008).

The lamina propria, underlying the intestinal epithelium, contains dendritic cells, macrophages, and natural killer cells, and they work to modulate the immune response when invading pathogens encounter the intestinal epithelium (Daneman and Rescigno, 2009). Macrophages are present at higher densities along the villi and are located closer to the epithelial cells than dendritic cells (Schulz et al., 2009). The infection of *T. gondii* at the mucous membranes of the lamina propria in the small intestine results in parasite invasion of different cell types, including dendritic cells, macrophages, and intestinal epithelial cells (Lambert and Barragan, 2010). Recently, the parasite effector GRA28 (dense granule protein) led to the upregulation of the macrophage receptor CCR7 generating the acquisition of dendritic cells-like migratory properties on macrophages impacting parasite dissemination in mice (ten Hoeve et al., 2022).

The impact of *T. gondii* infection and dissemination on the mucosa immune response has been well established by the study of intestinal non-immortalized cell line models (Snyder and Denkers, 2021). Nevertheless, those responses are hardly mimicked by the use of cell cultures because the immune components are not naturally integrated into the models, so the appeal of co-culture with immune cells is clear.

Among non-immortalized intestinal cells, primary cells (fully differentiated cells that can be isolated directly from the gut) and stem cells (fully or partially undifferentiated cells with the ability to differentiate into different specialized cells) can be distinguished. Primary cells can be cultured on different scaffolds including porous membranes and hydrogels to generate fully differentiated monolayers without stem cells, self-renewing monolayers with or without segregation of the stem and differentiated cell types, or proliferative monolayers over a layer of supportive feeder cells. The use of these cell types has relative advantages in the conservation of different cell properties in comparison to immortalized cells (Balimane and Chong, 2005). For example, functional, morphological, and structural properties like cell permeability changes (Takenaka et al., 2016), expression of proteins defining epithelial character (Anderle et al., 2004), and diversity on different epithelial cells. On the other hand, an important disadvantage of primary cells is the finite *in vitro* lifespan and decreased proliferation capacity, making these models inconvenient for addressing experimental questions that require long-term culturing. Specific media composition and scaffolding properties required to maintain stem cells *in vitro* are critically limiting factors.

Primary small intestinal epithelial cells have been used to study *T. gondii* infection with a focus primarily on the immune responses more than in the host-cell entry machinery. For example, the murine MODE-K and m-ICcl2 intestinal epithelial cells display altered chemokines production and proinflammatory responses after *T. gondii* infection (Mennechet et al., 2002; Gopal et al., 2011; Johnson et al., 2014). In addition, the use of a human fetal small intestinal epithelial cell line (FHs 74 Int) suggested that the activation of the inflammasome upon *T. gondii* infection induces the IL-1β secretion and parasite proliferation in the human small intestinal epithelial cells (Quan et al., 2018). These studies suggest that the modulation of gut inflammatory responses may serve as a mechanism to decrease epithelial cell responses and facilitate parasite dissemination and multiplication.

As described above, the intestinal epithelium is organized into proliferating crypts and differentiated villus. The crypts are the niche for intestinal stem cells (ISC), which are characterized by the expression of the specific marker Lgr5+. Paneth cells, also present at the crypt, help in regulating stem cell self-renewal to maintain intestinal homeostasis. The establishment of a 2D culture system to support ISC monolayer was successfully developed for mice (Liu et al., 2018), and those cells are the best documented ISCs in the study of intestinal infections (Mileto et al., 2020). The isolation of a subpopulation of ISCs from a less proliferative type called HOPX+ may serve a functional role in ISC-mediated regeneration after intestine damage and could control ISC proliferation (Stewart et al., 2021). *T. gondii* infection impairs the HOPX+ stem cell proliferation in the colonic mucosa in *in vivo* experiments (Saraav et al., 2021).

Although 2D cultures are simple, low-cost, scalable, and reproducible, the absence of a third dimension limits their ability to properly mimic the architecture of the tissue nor the cell-cell and cell-extracellular environment interactions. The following section discusses the development of 3D cell culture systems that closely mimics the *in vivo* conditions of the cellular microenvironment, opening new opportunities for achieving the complete *T. gondii* life cycle *in vitro*.

### 4.3 Intestinal organoids

The use of 2D-cultured cells has been extremely useful for understanding the mechanisms of infection of *T. gondii*. However, our understanding of the particular dynamics of infection happening at the intestinal epithelium has been hindered by the lack of appropriate models that recapitulate its complexity. The generation of three-dimensional (3D) cell cultures have opened new avenues for assessing direct pathogen-epithelium interaction in a more physiological context.

Organoids are tiny multicellular (3D) structures containing multiple-organ specific cells, derived from the self-renewal and self-organization potential of stem cells. They can be generated from adult stem cells (ASCs), embryonic stem cells (ESCs) or induced pluripotent stem cells (iPSCs) (Azar et al., 2021) provided the required growth and differentiation factors are present, in the presence of extracellular matrix components. Physiological resemblance of organoids to their originating organs is conserved at the levels of gene and protein expression, metabolic function, morphology, and the cell interactions occurring within the tissue (Weeber et al., 2015; Zhao et al., 2022). These features make organoids a promising opportunity to tackle the “physiological resemblance” gap between traditional cell cultures and *in vivo* animal experiments.

The first establishment of intestinal organoid culture was described by Sato and colleagues whereby the authors generated a mouse intestinal organoid from isolated crypts and single Lgr5 marker-positive stem cells using a basement membrane matrix and serum-free medium supplemented with Wnt agonist R-spondin 1, BMP signaling antagonist Noggin and epidermal growth factor (Sato et al., 2009; Sato et al., 2011b). This resulted in an *in vitro* model system called “mini-gut”. The generated crypt-villus structures consisted of stem cells and specialized epithelial cells i.e. enterocytes, paneth cells, goblet cells and enteroendocrine cells, lacking mesenchymal or hematopoietic lineages. Afterward, human intestinal organoids were established from human ESCs, human iPSCs, and primary human tissue (McCracken et al., 2011; Sato et al., 2011a; Spence et al., 2011). Later on, organoids from other species, including livestock animals (cattle, sheep, pig, horse, chicken) were developed (Powell and Behnke, 2017; Hamilton et al., 2018; Derricott et al., 2019; Mussard et al., 2020). Noteworthy, while intestinal cell models from mouse and human tissue are widely available, little is known about the differentiated cell types present in some farm animal intestinal epithelium (Derricott et al., 2019). Therefore, no cell lines are available to model their intestinal biology, rendering organoids as the only option to gain insight into their particular host-pathogen interaction dynamics.

Organoids have been widely used to study host-pathogen interactions within the intestinal environment, including bacteria, viruses, and parasites, where the presence of multiple cell types closely resembles the *in vivo* situation (Foulke-Abel et al., 2016; Dutta et al., 2017; Barrila et al., 2018; Duque-Correa et al., 2020; Smith et al., 2021). Over the past years, the field of cell culture bioengineering was revolutionized by the generation of organoids from different organisms and organs, many of which have been employed as exceptional *in vitro* systems to support the growth of several apicomplexan parasites (Ramírez-Flores et al., 2022). A caveat of intestinal organoids recreating *in vivo* biology is their inverted topology, where the basal side faces the surrounding culture media and the apical side faces the lumen, lying within the structure. This has required exploring methodologies to incorporate viable parasites directly into the luminal space, through organoid fragmentation, apical-out organoids or microinjection (Co et al., 2019; Smith et al., 2021).

The continuous division of intestinal epithelial stem cells in 2D and 3D cultures results in a fully differentiated and polarized epithelium that can be generated from different regions of the intestine, including duodenum jejunum, ileum, and colon. Specie-specific models provide opportunities for studying *T. gondii* infection, providing a specific window into its poorly understood sexual differentiation using feline-derived models. Additionally, the generation of intestinal organoids from non-felid organisms provides a potentially attractive tool to study *T. gondii* sexual differentiation, so long as the system allows *in vitro “*felinization” by means of chemically recreating the feline enteric environment. Next, we review some examples of the exciting uses of intestinal organoids to approach the biology of *T. gondii* within this tissue and discuss their applicability for developing new alternatives for studying sexual stages.

### 4.4 Intestinal organoids to study *T. gondii*

A robust protocol was described to establish and maintain stem-cell enriched organoid cultures and organoid-derived monolayers of human and mouse, but also from pig and chicken. These have proven suitable for *T. gondii* infection (Hares et al., 2020; Holthaus et al., 2021). For some species, particularly livestock, organoids are the only *in vitro* approach available to study *T. gondii*´s biology within their intestine as relevant cell lines are not at all available.

By microinjecting tachyzoites into the lumen of the closed organoid from bovine and porcine origin, researchers observed successful replication of the parasite 24 hours after infection (Derricott et al., 2019). Furthermore, Luu and colleagues developed an organoid derived from murine isolated crypts by the generation of a collagen-supported epithelial sheet with an exposed apical surface (Luu et al., 2019). The organoid was susceptible to the infection and supported replication and motility of *T. gondii* where parasites were observed in Paneth and goblet cells. Finally, after invasion and several cycles of replication within the cells, *T. gondii* egressed and invaded nearby cells. In addition to successful parasite’s lytic cycles in 3D intestinal cultures, changes in both the parasite and host cell transcriptomics have been mapped.

Transcriptome and proteome analyses from the small intestine epithelium of cats infected with *T. gondii* showed an increase of oocyst-wall genes and proteins (Ramakrishnan et al., 2019). In line with this, the transcriptome analysis of host epithelial cells from differentiated small intestinal organoids (from duodenal biopsy) after the infection (72 h) with the related coccidia *Cryptosporidium parvum* showed an increase in gene expression of multiple parasite oocyst-wall genes (Heo et al., 2018), highlighting the potential of this system for studying this phenomena *in vitro* using “felinized” intestinal organoids in the case of *T. gondii*. In fact, organoid-derived monolayers from human duodenal biopsy have been used to study the biology of *T. gondii* within the intestine. *T. gondii* infections of this model revealed a novel sequence of molecular events leading to epithelial barrier breakdown in this human primary tissue. These experiments uncovered that adenosine 3´,5´-cyclic monophosphate (cAMP)/protein kinase A (PKA) signaling affects the barrier breakdown by means of inducing TJ disruption in human intestinal epithelial cells (Holthaus et al., 2021).

Finally, co-culture of 3D cell models with immune cells such as dendritic cells was carried out in a stem cell-derived enteroids from mice intestinal epithelium infected with *T. gondii*, opening up the possibility of studying how the crosstalk with the epithelium influences dendritic cells’ function, and how the parasite alters these interactions (Hares et al., 2020). The co-culture of organoids directly with components of the immune response is also possible. Intestinal organoids generated from mice small intestine were co-cultured with recombinant murine cytokine IFN-γ, which mediates the death of Paneth cells after the infection with *T. gondii* by the control of the kinase complex mechanistic target of rapamycin (mTORC1) (Araujo et al., 2021).

Different methods using 3D intestinal systems are being developed to recreate more complex and physiological conditions, allowing the incorporation of microvasculature, microbiota, immune cells and microfluidics leading to a next step in 3D modeling with the organ-on-a-chip technology (Palikuqi et al., 2020; Bozzetti and Senger, 2022; Pimenta et al., 2022). The possibility of expansion of the organoid micro-architecture by addition of these factors may aid in understanding the impact of mechanical forces and biological elements on *T gondii´s* intestinal infection.

### 4.5 Intestine-on-a-chip

Organ-on-a-chip (OOAC) and multiorgan on a chip (MOC) represent the latest advancements in 3D culture technology. They are microscale devices that mimic the complex structure and function of organs by incorporating multiple cell types (OOAC) or multiple organ-specific chips (MOC). In addition, these devices incorporate extracellular matrix and physiological conditions such as fluid flow and mechanical forces allowing control of the cellular microenvironment and the mechanical dynamic of organs. Fluid concentration gradients (i.e. blood vessels), maintenance of geometry of the vasculature and epithelium, nutrient supplementation, metabolite emissions, mechanical stress, contractile properties, cell patterning and other external conditions can be controlled (Rajan et al., 2020; Baddal and Marrazzo, 2021). Overall, OOAC and MOC technologies are particularly useful when the biological question requires an increase in biological complexity of the system. It offers several advantages over traditional 2D and 3D cultures, including improved cell-to-cell matrix interaction, more physiologically relevant models, and real-time monitoring of cellular response. Over the past 10 years, intestinal-on-chip platforms have evolved from simple 2D cultures to include more comprehensive functionality, such as villi structures, intestinal peristalsis, oxygen gradients, and even immune systems and microbiome elements (May et al., 2017; Xiang et al., 2020). They have been used in the search of new clues in host-pathogen interactions including bacteria, virus, fungi, and parasites (Blutt et al., 2018; Grassart et al., 2019; Sunuwar et al., 2020; Tang et al., 2020). The use of OOAC in apicomplexan parasite research is only initiating. We briefly analyze here the early steps and possibilities that this new technology presents for the study of Apicomplexa focusing specifically on *T. gondii.*

Intestinal or gut OOACs have been designed, and revised protocols for their generation are currently available (Leung et al., 2022; Ramírez-Flores et al., 2022). Duodenum OOAC has been developed from human adult-derived intestinal organoids co-cultured with microvascular endothelial cells separated by a PDMS membrane (polydimethylsiloxane) (Kasendra et al., 2020). This system showed cytoarchitecture, cell-cell interactions, permeability parameters, and gene expression which more closely resemble those of human intestines than organoids do. This technology allows for more biologically faithful drug delivery and pharmacokinetic studies. These microfluidic systems can be used and adapted for research of enteric microorganisms, like *T. gondii*.

Remarkably, a long-lived and tube-shaped intestinal epithelial culture system has been reported by using crypt-like microcavities under flow, induced topography-guided self-organization of a functional epithelium with crypt- and villus-like domains similar to that observed *in vivo*. The culture system showed self-regeneration capacity and response to bacterial infection. Moreover, long-term parasite infection by infecting the mini-gut tubes with *C. parvum* was modeled. Live-cell microscopy showed that the tubular organoids support the entire life cycle and long-term growth of *C. parvum* without affecting tissue integrity, and immunofluorescence assays identified each asexual and sexual stage of *C. parvum* (Nikolaev et al., 2020).

Human intestinal crypts containing functional stem cells, derived from the jejunum region of the small intestine were integrated in an organ-on-chip devices including micro-physiological systems (MPSs) and co-cultured with immune cells (including neutrophils and NK cells) by the integration of an adjacent vascular lumen. The human intestinal tissue MPS supported the invasion, replication, and translocation of *T. gondii* beyond the epithelium (Humayun et al., 2022). *T. gondii* infection of MPSs stimulated a broad range of effector functions in neutrophils and natural killer cells–mediated cytokine production, which may play immunomodulatory roles in the host.

The microbiome presence in a human colon chip model was generated by co-incubation with human microbiome metabolites collected from PolyFermS continuous intestinal fermentation bioreactors. The authors of this study found that microbiome metabolites recapitulate species-specific tolerance in colon chips (Tovaglieri et al., 2019). Regarding oxygen levels, the manipulation of oxygen present in a MPSs from a mouse colon chip was incorporated by culturing under a hypoxia gradient created by flowing oxygenated medium through the basal channel (extracellular matrix-coated chips) while maintaining the entire chip in an anaerobic chamber filled with carbon dioxide and nitrogen gas (Gazzaniga et al., 2021). Finally, the intestinal epithelium is surrounded by smooth muscle layers, with the enteric neural system embedded to control intestinal peristalsis movements that control the motion within the intestinal cavity in a constant forward direction (Sinagoga and Wells, 2015). Artificial peristalsis was introduced into human colonic MPSs made from elastomeric polymer PDMS. The application of cyclic vacuum within the system induces strain and stretching of the porous membrane that recreates peristaltic-like motions, and the dependency of those mechanical forces strongly impacts pathogen invasion within the epithelium (Grassart et al., 2019). Future studies could incorporate biological factors such as specific microbiome, tissue oxygen levels, and peristaltic movements to better recapitulate the *in vivo* microenvironment of the intestine and to examine their influence on the biology of *T. gondii* both in intermediate hosts and in felids by exogenous “*felinization*” of the system.

### 4.6 Intestinal explants

In the late 60´, Browning and Trier described a successful method to culture human duodenojejunal junction sections that maintained normal morphology, proliferation, and absorption properties of the intestinal epithelium after 24 h (Browning and Trier, 1969). Explants are generated from fragments of native tissue, so they mimic closer than other systems the architecture and cellular composition (including other resident cells i.e immune cells and stromal cells) present in the intestine (reviewed in Randall et al., 2011). The explant culture of the gastrointestinal tract offers an *ex vivo* alternative to studying a wide range of intestinal infections. Noteworthy, however, the application of this technology has thus far been limited as its systematization is hampered by its inherent variability. In addition, explants have a limited lifespan undergoing rapid degeneration in a few days. Recently, however, approaches have been described that allow the maintenance of explant cultures for a prolonged time (Baydoun et al., 2017; Baydoun et al., 2020).

Despite its limitations, valuable data has been attained using these models pertaining to *T. gondii*´s biology. In particular, time-lapse imaging of murine intestinal explants infected with *T. gondii* has revealed parasite spreads through the lumen of the intestine while neutrophils are recruited to foci of infection and preferentially harbor parasites when compared to other leukocytes (Coombes et al., 2013). Mouse colon explants obtained from a severe combined immunodeficiency strain were able to survive and preserve the tissue morphology for 35 days, in the presence of microvilli, villi-like, and crypt-like structures, connective tissue with collagen, fibroblasts, and smooth muscle cells. This 3D model was able to support the growth of *C. parvum* for 27 days, resulting in the identification of previously unknown markers of lesions happening in the long-term infection process (Baydoun et al., 2017). The use of this model for the study of *T. gondii* remains to be explored.

A novel microphysiological system called intestinal explant barrier chip was reported using human and porcine colon explants. This system is based on a dynamic microfluidic microenvironment that extends tissue viability (Amirabadi et al., 2022). This *ex vivo* model revealed regional and interspecies differences in intestine properties since it has a more complex architecture that better preserves the qualities of the originating tissue. In the future, it would be possible to incorporate microbes like parasites to gain valuable insight into their biology in a species-specific manner.

## 5 Discussion

### 5.1 Challenges and opportunities for models to promote *T. gondii* sexual stages

Historically, *T. gondii* research has relied on 2D cell cultures and *in vivo* animal models. Animal models are expensive, time-consuming, and provide no or limited access to analyzing *in vivo* host-parasite interaction at the subcellular level. In addition, there are inherent ethical constraints and animal welfare concerns in working with animal models. In contrast, two-dimensional cultures of mammalian cells represent a cost-effective and convenient system for performing controlled, reproducible, infection studies integrating only a handful of variables. However, growing cells on flat surfaces pose structural constraints which lead to artificial morphology and altered behaviors, distinct from the physiological cell behavior within a tissue.

**Figure 2:**
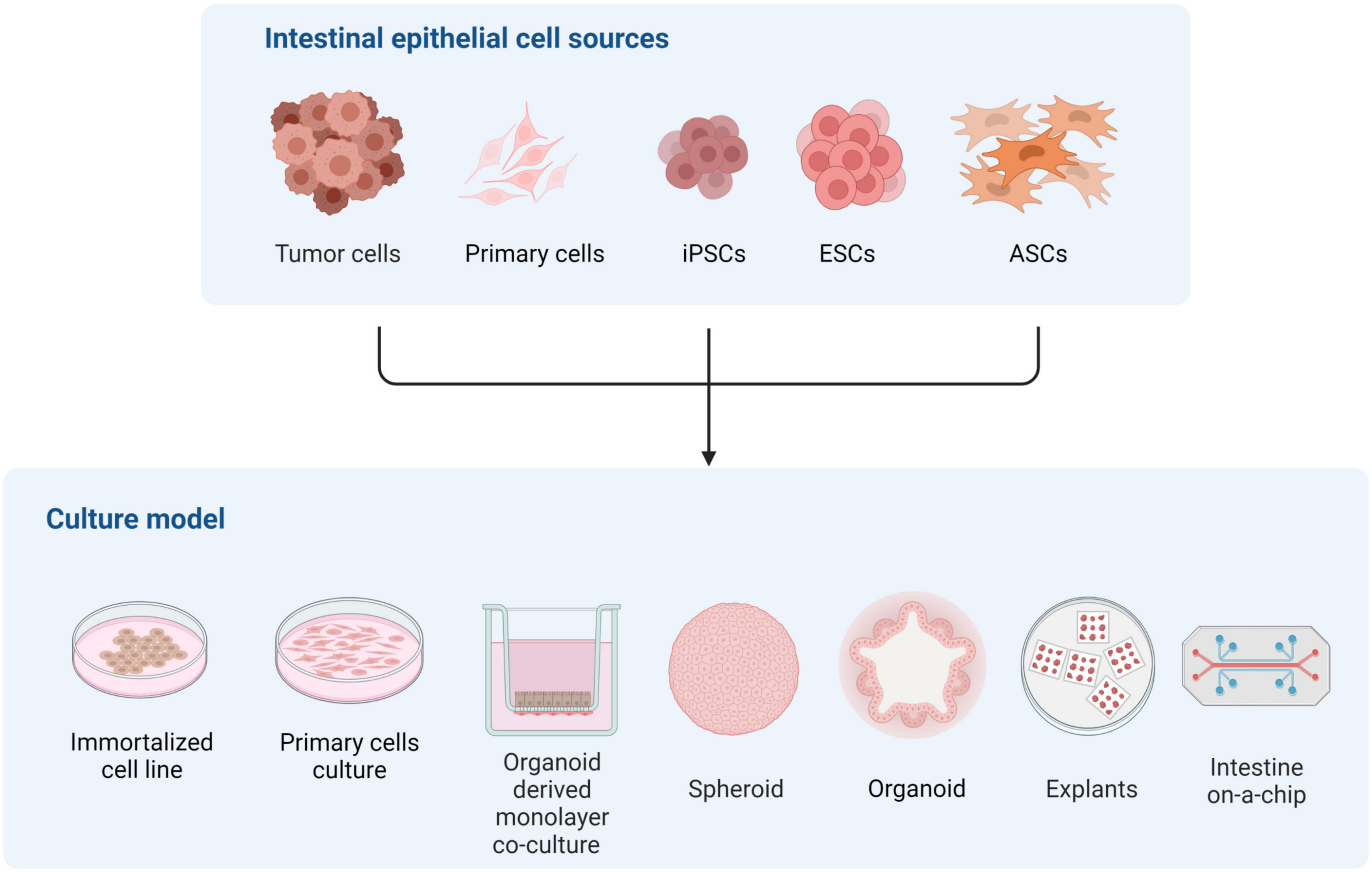
Representation of different *in vitro* models of the intestinal epithelium to study multiple aspects of *T. gondii* and related coccidian parasite biology.

The intestinal epithelium is an exceptional and unique model to study differentiation to sexual stages of many coccidia parasites, including *T. gondii*. However, recreating the complexity of the intestinal epithelium in an *in vitro* system is challenging, as evidenced by the different cell sources and culture approaches developed to tackle this task (**Figure 2**). Immortalized cell line such as Caco-2, which have been commonly used for studying *T. gondii* infection of the intestine (Barragan et al., 2005; Monroy, 2008; Briceño et al., 2016; Jones et al., 2017; Ross et al., 2019), can form polarized monolayers of enterocyte-like cells but lack the diversity of intestinal cell types. As a consequence, this model poorly recapitulates the physiology of the normal tissue. On the other hand, intestinal explants provide cellular diversity and natural tissue architecture, but rely heavily on animal-derived sample availability and display limited viability and ample variability, making it suitable for studying early infection and acutely occurring processes only.

Reconstructing host microenvironments including 3D tissue architecture, multicellular complexity, microbiota composition/localization, oxygen tension, transport processes, and biomechanical forces, are key to recreating *in vivo* pathogens’ biology *in vitro*. Many efforts have been pursued to develop a new generation of 3D *in vitro* models that more faithfully recapitulate these features, improving their predictive capabilities. In this context, organoids have arisen as alternatives to many of the traditional cell cultures since they are conformed by different intestinal cell types and recapitulate the 3D architecture and polarization of the cells in the intestine. Also, as organoids derive from stem cells, they can be expanded and maintained in culture for long periods of time, and cryopreserved. As an advantage to traditional cell lines, they can be cultured as 2D and 3D systems maintaining organ specificity and genome stability. Together, these particular properties turn the organoid culture model into a promising tool for unraveling *T. gondii* intestinal infection mechanisms both in intermediate and definitive hosts. Moreover, the possibility of recreating the feline intestinal environment in a human or murine organoid system is emerging as a valuable alternative to decipher *T. gondii* sexual differentiation (Di Genova et al., 2019).

Since *T. gondii* enters the intestine through the apical surface, and intact organoids have its apical side lying within the structure, using organoids to study *T. gondii* infection poses some difficulties. Nonetheless, several methodological approaches/strategies can be pursued for achieving *T. gondii* apical epithelial surface infection. Organoids can be fragmented to expose the apical surface and co-culture with pathogens (Luu et al., 2019; Holthaus et al., 2021). However, it is important to consider parasite invasion requirements and the effect on epithelial architecture since organoid structures are disturbed when this approach is selected. Another possibility is to microinject the pathogen directly into the organoid lumen, without altering organoid structure (Zhang et al., 2014; Wilson et al., 2015; Heo et al., 2018). Although this attractive strategy has been widely successful for bacteria and viruses, it is particularly challenging for parasites in general due to their comparatively larger size, though plausible for protozoa. The lumen condition (levels of oxygen and cell death accumulation) could also interfere with parasite survival and invasion. In this case, organoid-derived monolayers can be an excellent alternative method for culturing organoids in co-culture with parasites in a controlled and reproducible manner allowing direct access to the apical epithelium, maintaining the intestine’s cellular diversity. The use of a collagen-supported epithelial sheet model (Luu et al., 2019) or air-liquid interface cultures (Wilke et al., 2019), where cells are differentiated and polarized, have been explored as alternatives to study other complex parasite life cycles, like that of the *Cryptosporidium* species. There exists ample potential for applying these models in the future to the study of *T. gondii*´s biology within the intestine. Last but not least, organoids can also be culture with reverse polarity, where the basal layer is turned inward exposing the apical layer to the external media environment (Kakni et al., 2022). These inside-out organoids could provide important insights into *T. gondii* life cycle and its sexual reproduction.

Regardless of the success of *in vitro* models for shedding light onto *T. gondii*’s infection mechanisms (invasion, multiplication, and egress) (**Table 1**), data on the underlying events driving stage conversion remains limited largely due to the lack of adequate *in vitro* models that support the completion of the parasite life cycle. The *in vitro* systems used to study host-parasite interaction include organoids from pluripotent stem cells, organoid-derived monolayers, cell lines cultured in 3D silk-protein scaffolds (DeCicco RePass et al., 2017; Cardenas et al., 2020), hollow fiber technology (Morada et al., 2015) and colonic explants (Baydoun et al., 2017). DeCicco RePass et al., 2017 reported a novel bioengineered 3D human intestinal tissue model as a long-term infection system with the advantage of recreating oxygen gradient along the gastrointestinal tract. Altogether these innovative experimental platforms provide exciting alternatives on the quest for models that allow the full life cycle of *T. gondii* to be recreated *in vitro*. Improving these models using microfluidic approaches, incorporating peristaltic and flow conditions as well as co-culture with immune/stromal cells and gut microbiota are the next steps for a better *in vivo*-like environment *in vitro* recreation. Developing chemically “felinized” intestinal organoids, infected with bradyzoites and sporozoites as the sexual stage starting point will be crucial to gaining insight into the molecular underpinnings of *T. gondii*’s sexual cycle.

## Author contributions

FS and MF conceived the original idea and scheme of this revision. FS, SC, and RP created the figures and table. MF and MB-F contributed to funding acquisition. All authors contributed to reviewing the literature, wrote and approved the submitted version.
